# Deep learning-based thin-section MRI reconstruction improves tumour detection and delineation in pre- and post-treatment pituitary adenoma

**DOI:** 10.1038/s41598-021-00558-2

**Published:** 2021-10-29

**Authors:** Da Hyun Lee, Ji Eun Park, Yeo Kyung Nam, Joonsung Lee, Seonok Kim, Young-Hoon Kim, Ho Sung Kim

**Affiliations:** 1 Department of Radiology and Research Institute of Radiology, Asan Medical Centre, University of Ulsan College of Medicine, 43 Olympic-ro 88, Songpa-Gu, Seoul, 05505 Republic of Korea; 2 GE Healthcare Korea, Seoul, Republic of Korea; 3 Department of Clinical Epidemiology and Biostatistics, Asan Medical Centre, University of Ulsan College of Medicine, Seoul, Republic of Korea; 4 Department of Neurosurgery, Asan Medical Centre, University of Ulsan College of Medicine, Seoul, Republic of Korea

**Keywords:** Diagnostic markers, Pituitary tumours, Information technology

## Abstract

Even a tiny functioning pituitary adenoma could cause symptoms; hence, accurate diagnosis and treatment are crucial for management. However, it is difficult to diagnose a small pituitary adenoma using conventional MR sequence. Deep learning-based reconstruction (DLR) using magnetic resonance imaging (MRI) enables high-resolution thin-section imaging with noise reduction. In the present single-institution retrospective study of 201 patients, conducted between August 2019 and October 2020, we compared the performance of 1 mm DLR MRI with that of 3 mm routine MRI, using a combined imaging protocol to detect and delineate pituitary adenoma. Four readers assessed the adenomas in a pairwise fashion, and diagnostic performance and image preferences were compared between inexperienced and experienced readers. The signal-to-noise ratio (SNR) was quantitatively assessed. New detection of adenoma, achieved using 1 mm DLR MRI, was not visualised using 3 mm routine MRI (overall: 6.5% [13/201]). There was no significant difference depending on the experience of the readers in new detections. Readers preferred 1 mm DLR MRI over 3 mm routine MRI (overall superiority 56%) to delineate normal pituitary stalk and gland, with inexperienced readers more preferred 1 mm DLR MRI than experienced readers. The SNR of 1 mm DLR MRI was 1.25-fold higher than that of the 3 mm routine MRI. In conclusion, the 1 mm DLR MRI achieved higher sensitivity in the detection of pituitary adenoma and provided better delineation of normal pituitary gland than 3 mm routine MRI.

## Introduction

Pituitary adenoma with hormone excess, irrespective of size, sometimes requires accurate detection and surgical treatment^1,2^. In the management of growth hormone-releasing or adrenocorticotropic hormone (ACTH) secreting tumour, precise detection and localisation of adenoma is essential^3–5^. In addition, to selectively remove the tumour, avoid the risk of residual tumour, and preserve functioning normal tissue, surgeons must precisely distinguish normal pituitary stalk and gland from pituitary adenoma^6,7^. Therefore, dedicated high-resolution magnetic resonance imaging (MRI) protocols have been proposed to detect pituitary adenoma and accurately guide surgical removal^8–11^.

In clinical practice, 3 mm slice thickness MRI is routinely used^12–14^. However, the thickened slices lead to the partial volume averaging effect^15,16^, which may result in insufficient delineation of the tumour and adjacent anatomical structures. Reducing the slice thickness has been difficult because it decreases signal-to-noise ratio (SNR), degrades image quality^17^, and decreases diagnostic accuracy^18,19^. Recently, deep learning-based reconstruction (DLR) was introduced to address this trade-off between SNR and spatial resolution. Denoising uses unique pattern recognition to isolate noise and low resolution to reconstruct the ideal object image^20^. As such, DLR could be used to better detect pituitary adenoma and delineate normal pituitary stalk and gland from tumour tissue.

We hypothesised that 1 mm slice thickness DLR MRI may be superior to 3 mm slice thickness MRI in the detection of adenoma and delineation of pituitary stalk and normal gland in patients with pituitary adenoma as it provides both thin sectioned as well as denoised images. The purpose of the present study was to compare the diagnostic performance and image preference in the paired 1 mm DLR MRI and 3 mm routine MRI for clinical translation and application of DLR MRI. Also, we expected it would be helpful for inexperienced radiologists and clinicians who are not familiar with DLR images and do not have preference biases. Thus, we additionally compared both diagnostic performances and image preference between inexperienced and experienced radiologists.

## Materials and methods

### Study population

**Figure 1:**
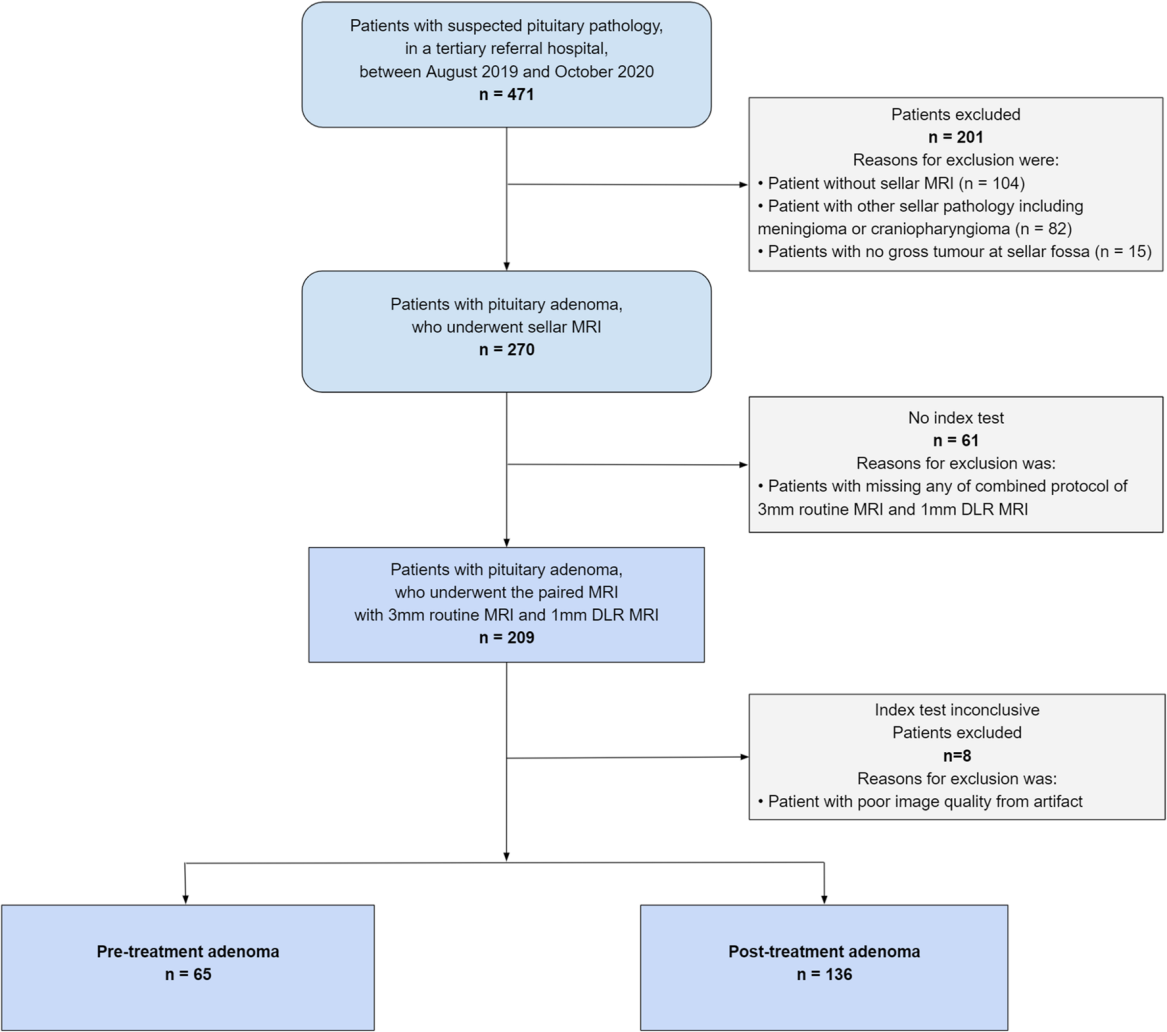
Flow chart of the patients included in the study. *MRI* magnetic resonance imaging.

This retrospective clinical study followed the 2015 guidelines of the Standards for Reporting of Diagnostic Accuracy statement. A total of 471 patients with suspected pituitary pathology were retrospectively reviewed at a tertiary referral hospital—Asan Medical Centre (Seoul, Republic of Korea)—between August 1, 2019, and October 30, 2020. Patients with the following inclusion criteria were recruited: (1) clinically suspected cases of pituitary adenoma or those who had undergone prior surgery to remove pituitary adenoma; and (2) they had undergone sellar MRI. Patients were excluded if: (1) they did not undergo the sellar MRI (n = 104); (2) they had other sellar diseases, including meningioma or craniopharyngioma (n = 82); (3) they had no gross tumour at the sellar fossa (n = 15); (4) they were missing any part of the combined protocol of 3 mm routine MRI and 1 mm DLR MRI (n = 61); and (5) they showed image artifacts due to aneurysm clips and dental hardware (n = 8). Finally, 201 patients with pituitary adenoma were included. Among them, 65 were pre-treatment and 136 had post-treatment adenoma. Figure 1 shows a flowchart of patient inclusion.

### MRI acquisition protocol

MRI was performed using a single 3.0-T machine (SignaTM Architect; GE Healthcare, Waukesha, WI) with a 48-channel head coil. A gadolinium-based contrast agent was intravenously injected before the examination at 0.1 mmol/kg of body weight using a power injector (Spectris; Medrad, Pittsburgh, PA). All injections were followed by a saline flush of up to approximately 30 mL. The first post-contrast image acquisition was carried out 3 min after injection.

The MRI protocol included the following: sagittal T1-weighted imaging (WI), coronal T2-WI, coronal T1-WI, sagittal/axial contrast-enhanced T1-WI, 3 mm slice-thickness routine MRI coronal contrast-enhanced T1-WI (3 mm routine MRI), and 1 mm slice-thickness coronal contrast-enhanced T1-WI with deep-learning reconstruction (1 mm DLR MRI). The detailed scan parameters of the 3 mm routine MRI were as follows: contrast-enhanced T1-weighted imaging; repetition time (TR)/echo time (TE), 500/13 ms; flip angle, 90°; field of view, 180 × 180 mm; matrix, 260 × 260; slice thickness, 3 mm with no gap; scan time, 3 min. Scan parameters of the 1 mm DLR MRI were as follows: coronal contrast-enhanced T1-weighted imaging; TR/TE, 698/16 ms; flip angle, 90°; field of view, 180 × 180 mm; matrix, 320 × 260; slice thickness, 1 mm with no gap; scan time, 4 min 8 s).

The MRI sequences were captured in two different orders following injection of contrast material: 3 mm routine MRI first (n = 50) or 1 mm DLR MRI first (n = 151).

### Deep learning-based reconstruction (DLR)

Commercially available AIRTM Recon DL (GE Healthcare, Waukesha, WI)^21^ was used to denoise 1 mm thin slice MRI. This software uses a deep convolutional neural network (CNN)-based algorithm embedded in the MRI reconstruction pipeline^20^. The algorithm takes raw k-space data as its input and generates high fidelity images as its output. The deep convolution neural network contains 4.4 million trainable parameters in approximately 10,000 kernels, which was trained using pairs of images representing near-perfect and conventional MRI images. Compared with conventional machine learning image reconstruction, the deep learning algorithm suppresses Gibbs ringing and truncation artifact and provides higher spatial resolution with more highly defined edges^20,21^. The software provides a user-specified denoising level. In the present study, a 70% noise reduction factor was chosen. The detailed network design and performance in phantom images is shown in the white paper^20^. The time required for image reconstruction was about 2 min.

### Image analysis

#### Training and blinding

There were four readers, two experienced readers (J.E.P and H.S.K., with 9 and 24 years of experience in neuroradiology, respectively) and two inexperienced readers (Y.K.N., and D.H.L, both with 1 year of experience in neuroradiology). The definition of in experienced readers was that neither of the two inexperienced readers had any experience in 1 mm DLR MRI, although they had experience in 3 mm routine MRI. The 20 cases for the training session were randomly selected from the study population. The training session was for all four readers. In this session, the readers reviewed 20 cases and received feedback on their detection of pituitary adenoma and delineation of normal pituitary stalk and gland. If the lesion was detected by experienced readers and not by inexperienced readers, the inexperienced readers were advised and learned to detect new lesions. After the training session a break of two weeks was taken for wash-out, following which the four readers reviewed 402 paired MR images from 201 patients (1 mm DLR and 3 mm routine MRI). During image analysis, the readers were blinded to the patients’ clinical and radiological information, as well as to interpretations by on-site investigators. Each reader evaluated all patient images separately and independently on a multimonitor workstation.

#### Reference standard for tumour detection and image preference

Reference standards were prepared for tumour detection and image preference after a one month wash-out period for image analyses. First, the consensus reference standard for presence of residual or recurrent tumour and newly detected tumour was established by two experienced readers (J.E.P., and H.S.K) and two neurosurgeons (Y.H.K. and J.H.K., with 10 and 25 years of experience in neurosurgery, respectively). They utilised all available imaging resources, clinical histories, laboratory findings, operative records, and pathological reports. A recurrence was confirmed via long-term endocrinologic follow-up examinations in patients with functioning pituitary adenomas. For non-functioning adenomas, recurrence was diagnosed when the patients required subsequent secondary management, including radiation therapy.

Second, to compare an image preference of the inexperienced readers for delineating the pituitary gland and stalk, a consensus on image preference was reached by the two experienced readers (J.E.P., and H.S.K). Before reaching consensus, the agreement between the experienced readers for imaging preference was substantial (κ = 0.76; 95% CI, 0.63–0.89) and the number of disagreements between the experienced readers was 32 cases (15.9% [32/201]).

#### New detection of pituitary adenoma

The readers recorded new detection on 1 mm DLR MRI that was not visualised on 3 mm routine MRI, as well as new detection on 3 mm routine MRI that was not visualised on 1 mm DLR MRI. All newly detected lesions were confirmed as pituitary adenoma based on the reference standard.

#### Image preference for delineation of normal pituitary stalk and gland

The image analysis was conducted with a matched-pair design, comparing the images while blinding the reader to clinical information. The readers expressed their preference between 3 mm routine MRI and 1 mm DLR MRI on a 3-point scale (1 = preferred 3 mm routine MRI, 2 = equal, 3 = preferred 1 mm DLR MRI). The readers evaluated their preference based on their ability to delineate the margin between the tumour and the normal gland and anatomic structures, including the pituitary stalk and normal pituitary gland. Joint assessment of pituitary stalk and gland was performed as these structures should be minimally manipulated during treatment to prevent hypopituitarism and avoid life-long hormone replacement after surgery^22^ or radiotherapy^23^. In addition, considering that the detectability of pituitary adenoma may be affected by the timing of contrast injection^24^, a subgroup analysis was performed for the imaging preference according to the order of sequences between 3 mm routine MRI and 1 mm DLR MRI.

### Quantitative evaluation of image quality

In 30 randomly selected patients, the SNR and contrast-to-noise ratio (CNR) between the pituitary gland and brain parenchyma were calculated for 1 mm DLR MRI and 3 mm routine MRI. Regions of interest (ROIs) were placed in the background, brain parenchyma, and normal pituitary gland. The standard deviation of the background ROI signal intensity was considered noise. The mean signal intensity was measured in each annotated ROI. The ROI of brain parenchyma was mainly located in the white matter to avoid enhancement-increasing structures such as blood vessels. The SNR of the pituitary gland was calculated as the mean signal intensity of the pituitary gland divided by noise. The CNR between the pituitary gland and brain parenchyma was defined as the absolute difference in mean signal intensity between the two tissues divided by the noise.

### Statistical analysis

The sensitivity, specificity, and accuracy of detection of residual tumour and newly detected tumour that were not found in previous exams were assessed across all cases based on the reference standard.

The distribution of preference between 3 mm routine MRI and 1 mm DLR MRI was first compared between experienced and inexperienced readers using the McNemar test with a Wilson 95% confidence interval (CI), which is an extension of normal approximation in that the actual coverage probability is closer to the nominal value^25^. The percentage of preference between the 3 mm routine MRI and 1 mm DLR MRI, according to the order of the sequences, was then assessed and compared using Fisher’s exact test.

Inter-reader agreement for the preference between 3 mm routine MRI and 1 mm DLR MRI was assessed using κ statistics. Agreement was classified as κ values < 0 indicating no agreement; 0–0.20, slight agreement; 0.21–0.40, fair agreement; 0.41–0.60, moderate agreement; 0.61–0.80, substantial agreement; and 0.81–1, almost perfect agreement.

The SNR and CNR of the 3 mm routine MRI and 1 mm DLR MRI were compared using the paired t-test. The SNR and CNR ratios were calculated using the one-sample t-test.

All statistical tests were conducted at a significance level of *p* < 0.05. Statistical analyses were performed by a biostatistician (S.O.K., with 10 years of experience in biostatistics) using SAS software (SAS Institute, North Carolina, US) and R software version 3.6.1 (R Core Team, Vienna, Austria).

### Ethics approval

All procedures performed in this study complied with both the US Health Insurance Portability and Accountability Act (HIPAA) regulations and the Declaration of Helsinki. This retrospective study was approved by the Institutional Review Board of Asan Medical Centre (approval number: 2020-1833). The requirement for written informed consent was waived by the Clinical Research Review Committee of Asan Medical Centre.

## Results

### Patient demographics

Patient characteristics are shown in Table 1. A total of 201 patients were enrolled (mean age ± standard deviation, 52 ± 14 years; 113 women [56%]). Sixty-five patients had pre-treatment pituitary adenoma and 136 had post-treatment adenoma. The median interval between the initial surgery and MRI scan was 23 months (interquartile range, 82 months; range, 1 day to 82 months).

**Table 1:** Demographic and clinical variables of the study patients.

Characteristics	Value
Total number^a^	201
Mean age (years ± standard deviation)^b^	52 ± 14
Sex (male:female)^a^	88:113
**Treatment stage**^a^	
Pre-treatment	65 (32.3)
Post-treatment	136 (67.7)
Open surgery	5
TSA	104
Radiation therapy	7
Stereotactic radiosurgery	20
**Tumour type**^a^	
Non-functioning adenoma	117 (58.2)
Functioning adenoma	84 (41.8)
Prolactin	58
Growth hormone	15
ACTH	11
Median time between the initial surgery and MRI examination (months)^c^	23 ± 82

*TSA* transsphenoidal surgery, *ACTH* adrenocorticotropic hormone, *MRI* magnetic resonance imaging.

^a^Data are expressed as count, with percentage in parentheses.

^b^Data are expressed as mean, with standard deviation.

^c^Data are expressed as median, with interquartile range.

There were 84 functioning pituitary adenomas (42%), which included 58 prolactinomas (29%), 15 growth hormone-secreting pituitary adenomas (7%), and 11 ACTH-releasing pituitary adenomas (5%).

### Accuracy of two inexperienced readers in residual tumour detection

Among 136 post-treatment patients, residual or recurrent tumours were found in 74 (54%). The inexperienced readers noted no significant differences in the detection of residual or recurrent tumours (Table 2). There was no difference of in detectability according to the order of the sequence.

**Table 2:** Diagnostic performance of residual or recurrent tumour.

	Sensitivity (%) (95% CI)	Specificity (%) (95% CI)	Accuracy (%) (95% CI)
Inexperienced reader 1	89 (80–96) [66/74]	89 (78–95) [55/62]	89 (82–94) [121/136]
Inexperienced reader 2	91 (81–95) [67/74]	81 (69–90) [50/62]	86 (79–91) [117/136]

*95% CI* 95% confidence interval.

### Comparison of new detection of pituitary adenoma

Newly detected adenomas (overall 6.5% [13/201];four patients with pre-treatment adenoma and nine with post-treatment adenoma) on the 1 mm DLR MRI were reported, which were not visualised on the 3 mm routine MRI. There was no significant difference depending on the experience of the readers, although one inexperienced reader reported significantly more lesions on 1 mm DLR MRI than in 3 mm routine MRI (*p* < 0.001). The two experienced readers reported 12 (5.9% [12/201]) and 14 new detections (6.9% [14/201]), respectively, of pituitary adenoma on 1 mm DLR MRI that were not visible on 3 mm routine MRI. The two inexperienced readers reported 11 (5.4% [11/201]) and 30 (14.9% [30/201]) new detections on 1 mm DLR MRI, respectively (Table 3).

**Table 3:** Diagnostic performance of newly detected pituitary adenoma on 1 mm DLR MRI, which was not visualised on 3 mm routine MRI.

	Sensitivity (%) (95% CI)	Specificity (%) (95% CI)	Accuracy (%) (95% CI)
Inexperienced reader 1	46 (23–71) [6/13]	97 (94–99) [183/188]	94 (90–98) [189/201]
Inexperienced reader 2	77 (50–92) [10/13]	89 (84–93) [168/188]	89 (84–93) [178/201]

95% CI = 95% confidence interval, 1 mm DLR MRI = 1 mm slice thickness MRI with deep learning-based reconstruction, 3 mm routine MRI = 3 mm slice thickness MRI.

**Figure 2:**
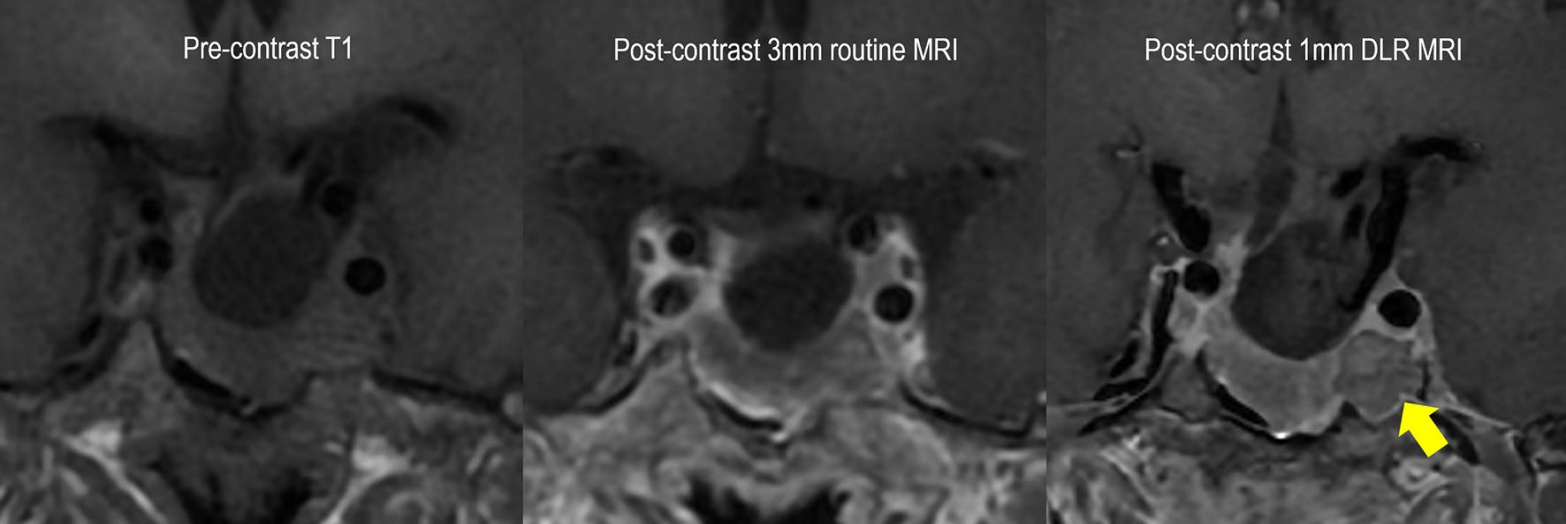
Representative case of postoperative state of pituitary adenoma with a residual lesion. A 52-year-old female patient underwent transsphenoidal resection of a non-functioning pituitary adenoma and underwent follow-up MRI 3 months later. She had residual tumour measuring 14 mm in the left cavernous sinus, which was not delineated on 3 mm MRI. However, 1 mm DLR shows the residual tumour clearly with improved sharpness of the edges. MRI = magnetic resonance imaging, 1 mm DLR = 1 mm slice thickness MRI with deep learning-based reconstruction.

Most of the newly detected lesions on 1 mm DLR MRI were in the postoperative status group (69%; 9/13), with underlying severe architectural distortion. None of them showed suspected recurrence/residual tumour on 3 mm routine MRI. Figure 2 shows a representative case. The delineation margin is conspicuous on the 1 mm DLR MRI, and the high contrast between the tumour and adjacent tissue is shown.

**Figure 3:**
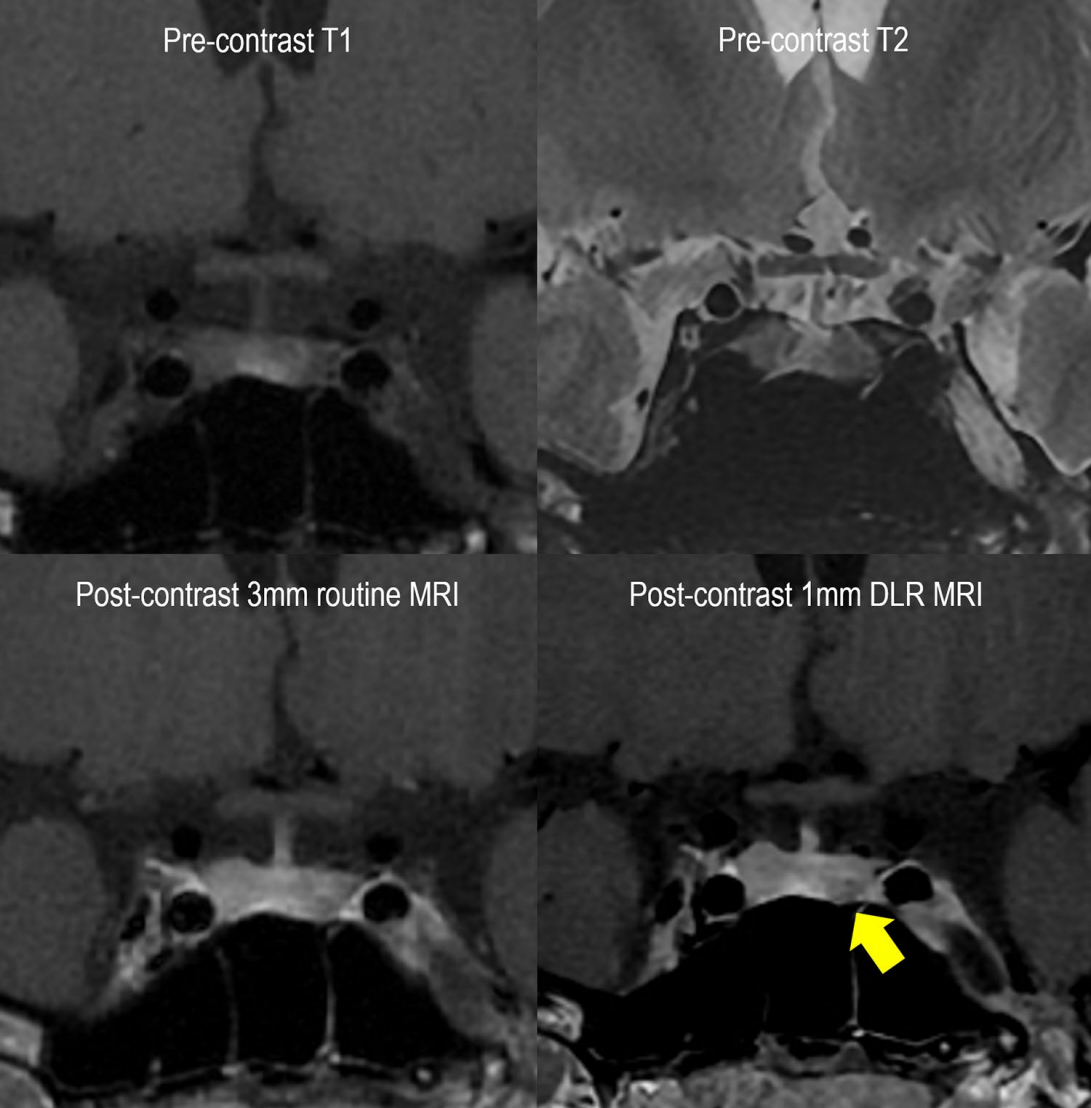
Representative case of newly detected pituitary microadenoma. A 19-year-old female patient had amenorrhea and underwent MRI as an initial evaluation. There is no grossly defined lesion on T2WI, T1WI, and 3 mm routine MRI. However, 1 mm DLR shows minute pituitary adenoma in the left inferior pituitary (arrow). MRI = magnetic resonance imaging, T2WI = T2-weighted image, T1WI = T1-weighted image, 3 mm routine MRI = 3 mm slice thickness MRI, 1 mm DLR = 1 mm slice thickness MRI with deep learning-based reconstruction.

The other four newly detected lesions on 1 mm DLR MRI were in pre-treatment patients. Figure 3 shows a representative case. A microadenoma measuring less than 5 mm is depicted on 1 mm DLR MRI. However, it was not discernible on 3 mm routine MRI and other sequences of T1- and T2-weighted imaging.

### Readers’ imaging preference for delineating normal pituitary stalk and gland

The results of imaging preference are shown in Table 4. All readers preferred 1 mm DLR MRI over 3 mm routine MRI to delineate normal pituitary stalk and gland. The experienced readers preferred 1 mm DLR MRI to 3 mm routine MRI in 56% of cases for delineating normal pituitary stalk and gland.

**Table 4:** Readers’ imaging preference for delineating normal pituitary stalk and gland.

	Prefer 1 mm DLR MRI	Equal preference	Prefer 3 mm routine MRI	*p* value^a^	*p* value^b^
Overall (n = 201)					
Consensus between the experienced readers	113 (56%)	83 (41%)	5 (3%)	Ref	
Inexperienced reader 1	138 (69%)	55 (27%)	8 (4%)	**< 0.001**	
Inexperienced reader 2	164 (82%)	23 (11%)	14 (7%)	**< 0.001**^**a**^	
Order of the sequences with contrast enhancement: 3 mm routine MRI first (n = 50) versus 1 mm DLR MRI first (n = 151)					
Consensus between the experienced readers	32 (64%) versus 81 (53%)	17 (34%) versus 66 (44%)	1 (2%) versus 4 (3%)		0.42^b^
Inexperienced reader 1	37 (74%) versus 101 (67%)	12 (24%) versus 43 (28%)	1 (2%) versus 7 (5%)		0.55^b^
Inexperienced reader 2	39 (78%) versus 125 (83%)	7 (14%) versus 16 (11%)	4 (8%) versus 10 (7%)		0.75^b^

Bold values used for emphasizing only values below p value < 0.05.

Data are expressed as count with percentage in parentheses.

1 mm DLR MRI = 1 mm slice thickness MRI with deep learning-based reconstruction, 3 mm routine MRI = 3 mm slice thickness MRI.

^a^ *p* value refers to the statistical difference in imaging preference between expert consensus (reference) and each reader.

^b^*p* value refers to the statistical difference in imaging preference according to the order of the sequences with contrast enhancement.

**Figure 4:**
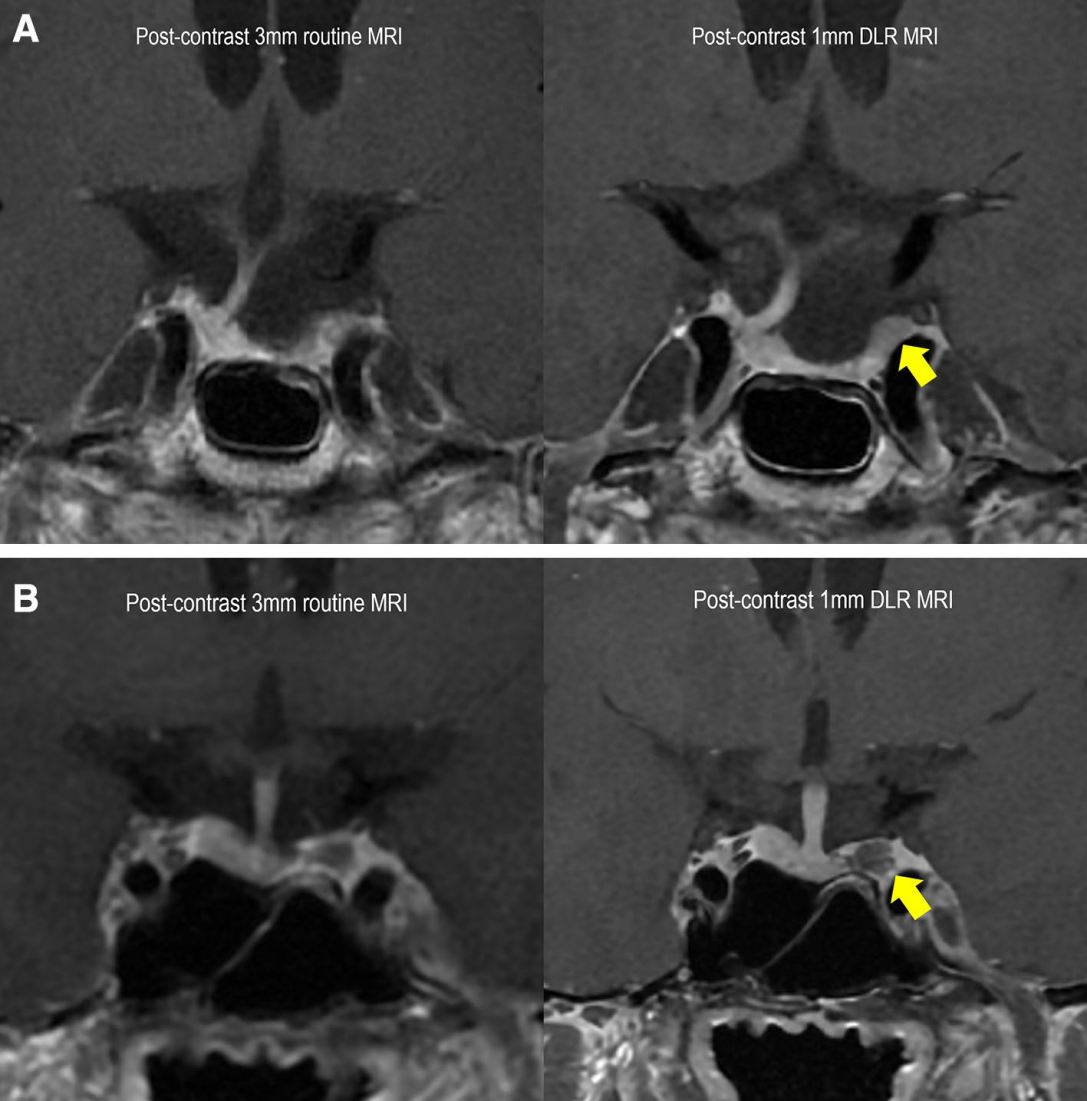
Representative case of improved delineation of pituitary gland and stalk. (**A**) A 65-year-old male patient underwent transsphenoidal resection of non-functioning pituitary adenoma and follow-up MRI 27 months later. The 1 mm DLR shows superiority in the detection of residual tumour delineation of normal pituitary gland and stalk, compared to the 3 mm routine MRI. (**B**) A 43-year-old male patient underwent transsphenoidal resection of prolactinoma and underwent follow-up MRI 41 months later. The 1 mm DLR was superior to 3 mm routine MRI in the detection of residual tumour, as well as in the delineation of normal pituitary gland and stalk. MRI = magnetic resonance imaging, 1 mm DLR = 1 mm slice thickness MRI with deep learning-based reconstruction.

The two inexperienced readers preferred the 1 mm DLR MRI to the 3 mm routine MRI even more strongly than the experienced readers (69% in the inexperienced reader 1, *p* < 0.001; 82% in the inexperienced reader 2, *p* < 0.001). Figure 4 shows an example of how 1 mm DLR MRI is superior to 3 mm routine MRI for delineating normal pituitary stalk and gland. The agreement between the two inexperienced readers was fair (κ = 0.35; 95% CI, 0.23–0.46), and the inexperienced reader 2 strongly favoured 1 mm DLR MRI over 3 mm routine MRI compared to the inexperienced reader 1.

The difference in imaging preference depending on the order of the sequences with contrast enhancement was further tested (also in Table 4). There was no significant difference in imaging preference depending on the order of sequences in the consensus between the two experienced readers (*p* = 0.42 by chi-squared test), and the inexperienced readers (inexperienced reader 1 [*p* = 0.55] and inexperienced reader 2 [*p* = 0.75]). All the readers preferred 1 mm DLR MRI.

### Quantitative comparison of image quality

A comparison of image quality is shown in Table 5. The SNR between the normal pituitary gland and brain parenchyma on 1 mm DLR MRI was 1.25-times higher than that on 3 mm routine MRI (mean SNR ± standard deviation, 253.9 ± 91.9 in 3 mm routine MRI vs. 308.9 ± 111.3 with 1 mm DLR MRI; *p* < 0.001). Similarly, the CNR between the normal pituitary gland and brain parenchyma were 1.35-times higher in 1 mm DLR MRI than in 3 mm routine MRI (mean CNR ± standard deviation, 133.5 ± 62.4 in 3 mm routine MRI vs. 154.1 ± 59.7 in 1 mm DLR MRI; *p* < 0.001). In detail, the subgroup analysis shows similar tendency in the recurrent lesions (n = 16) and newly diagnosed tumour (n = 14).

**Table 5:** Comparison of Image Quality between 1 mm DLR MRI and 3 mm Routine MRI.

	1 mm DLR	3 mm routine MRI	*p* value ^a^
**Overall (n = 30)**			
SNR (mean ± standard deviation)	308.9 ± 111.3	253.9 ± 91.9	**0.04**
CNR ^b^ (mean ± standard deviation)	154.1 ± 59.7	133.5 ± 62.4	0.19
SNR ratio	1.25 ± 0.34		**< 0.001**
CNR ratio	1.35 ± 0.85		**< *0.001***
**Post-treatment adenoma (n = 16)**			
SNR (mean ± standard deviation)	283.3 ± 97.4	*238.0* ± *79.7*	**0.01**
CNR ^b^ (mean ± standard deviation)	136.7 ± 53.2	125.4 ± 49.4	0.19
SNR ratio	1.22 ± 0.31		**< 0.001**
CNR ratio	1.15 ± 0.36		**< 0.001**
**Pre-treatment adenoma (n = 14)**			
SNR (mean ± standard deviation)	338.1 ± 122.2	272.3 ± 104.2	**0.01**
CNR ^b^ (mean ± standard deviation)	174.0 ± 62.4	142.8 ± 75.5	0.11
SNR ratio	1.29 ± 0.35		**< 0.001**
CNR ratio	1.57 ± 1.11		**< 0.001**

Bold values used for emphasizing only values below p value < 0.05.

1 mm DLR MRI = 1 mm slice thickness MRI with deep learning-based reconstruction, 3 mm routine MRI = 3 mm slice thickness MRI, SNR = signal-to-noise ratio, CNR = contrast-to-noise ratio.

^a^*p* value was calculated using paired contrast enhancement.

^b^CNR of tumour was measured in reference to the brain parenchyma (CNR of brain parenchyma) and normal pituitary gland (CNR of normal pituitary gland).

## Discussion

The present study demonstrated that 1 mm DLR MRI provides thin slice images that increase the sensitivity for detecting pituitary microadenoma and small recurrent/residual tumour after initial surgery. In 6.5% of cases (13/201), adenoma was newly detected on 1 mm DLR MRI that was not visualised on 3 mm routine MRI. The readers preferred 1 mm DLR MRI over 3 mm routine MRI for delineating normal pituitary stalk and gland. The inexperienced readers preferred 1 mm DLR MRI more strongly than the experienced readers. On quantitative assessment, the 1 mm DLR MRI showed higher SNR and CNR than the 3 mm routine MRI. Based on our results, 1 mm DLR MRI is more valuable clinically than 3 mm routine MRI because it has higher sensitivity for detecting pituitary adenoma and allows better delineation of normal pituitary gland in pre- and postoperative adenoma, facilitating accurate guidance during surgery.

The sellar and juxta-sellar regions are made up of various structures such as pituitary gland, as well as cerebrospinal fluid, cavernous sinus, sphenoid sinus, and bone. Particularly in the postoperative state of pituitary adenoma, granulation tissue or old blood products are likely to cause confusion in the diagnosis^26–28^. Deep learning-based denoising collectively removes noise and highlights meaningful signals such as edge sharpness^29^. In a recent study into postoperative pituitary adenoma^30^, 1 mm DLR MRI could diagnose residual tumour as reliably as 3 mm routine MRI. The present study found small but clinically meaningful detection of new adenoma using 1 mm DLR MRI, based on the consensus of both experienced readers and neurosurgeons on a reference standard. This further demonstrated the value of 1 mm DLR MRI. Detection of functioning pituitary adenoma at early stages is particularly important in Cushing’s disease^31^ and acromegaly.

As pituitary adenoma grows, the surrounding normal pituitary tissue forms a pseudo-capsule around the tumour^32^. Exact localisation of the pituitary adenoma and distinction from normal pituitary tissue enables selective tumour resection and prevents recurrence^33^. Moreover, the pituitary stalk is a thin connective bundle that carries axons from hypophysis nuclei and portal venous plexus to the pituitary gland. Therefore, clear visualisation of the pituitary stalk may allow surgeons to avoid postoperative hormone dysfunction, such as diabetes insipidus or anterior pituitary hormone deficit caused by possible pituitary axis injury^28,34,35^. Therefore, detailed further research is needed to compare the interpretations of experienced readers with those of inexperienced readers regarding morphological assessment for delineating the pituitary stalk and residual lesion, and to correlate the delineation of pituitary adenoma with clinical outcome.

The inexperienced readers preferred 1 mm DLR MRI more strongly than the experienced readers. The experienced readers showed substantial agreement while the inexperienced readers showed fair agreement in image preference, and one inexperienced reader strongly preferred 1 mm DLR MRI over 3 mm routine MRI. The detection accuracy was better on 1 mm DLR MRI. This indicates 1 mm DLR MRI images provide not only ‘good looking’ images but clinically meaningful detection for neurosurgeons and endocrinologists who have not seen DLR images before. This study did not compare the performance of the inexperienced readers without the DL model since its main purpose was the clinical translation and application of DLR in pituitary imaging. The superiority of the image quality and diagnostic performance of the 1 mm DLR images to those of the 1 mm non-DLR images was demonstrated in a previous feasibility study^30^.

Quantitative analysis confirmed that DLR increased SNR (1.25 times) and CNR (1.35 times). In previous studies, DLR has improved the quantitative SNR^36^ and visual quality of MRI^20^, which might help clinical diagnosis and management. The DLR algorithm used in the present study was built into a MR machine. The image processing time is relatively short, and the user can easily modulate the level of denoising^21^. If the algorithm included a step determining the different denoising level for different image slice, SNR deterioration from the artifact of metal or cerebrospinal fluid flow could be further minimised.

Some limitations should be addressed. Firstly, the present study was retrospective, the reference standard was based on clinico-radiological consensus, not all diagnoses were confirmed pathologically because most patients had non-functioning adenoma or well-controlled functioning adenoma. For future studies, a prospective observation of newly detected lesions may be necessary. Second, negative pituitary MRI scans were not included in the study design, which is a major limitation of the study. A future study design would be to include all MRI pituitary studies followed by blinded readers assessing for the presence or absence of a pituitary adenoma. Third, we sampled 20 cases from the study population for reader training, and the readers’ performance might have been biased due to recall bias and because the two-week wash-out period might have been insufficient. Fourth, dynamic contrast-enhanced T1-weighted imaging is a gold standard diagnostic tool for pituitary adenoma, especially for pituitary microadenoma. Currently, there is no feasible deep learning-based reconstruction algorithm for dynamic contrast-enhanced MRI. Technical advances are needed in this field. Furthermore, future studies directed to radiomics based on high-dimensional imaging data is feasible using DLR detected pituitary adenoma. Clinical questions of ‘do radiomics features of deep learning reconstruction differ from those of conventional images?’, ‘do radiomic features distinguish recurrent tumours from post-operative changes?’, ‘do radiomic features in pituitary adenoma have a correlation with a specific hormone?’, can be addressed.

In summary, 1 mm thin-slice MRI with DLR achieved higher sensitivity for detecting pituitary adenoma and provided better delineation of normal pituitary gland than a 3 mm routine MRI. Therefore, 1 mm DLR MRI most likely is more beneficial clinically, as thin-slice MRI applying deep learning-based denoising would contribute to proper treatment and management of pituitary adenoma.

## Data Availability

The datasets generated during and/or analysed in the current study can be obtained from the corresponding author on reasonable request.

## References

[1] Casanueva FF (2006). Guidelines of the Pituitary Society for the diagnosis and management of prolactinomas. Clin. Endocrinol..

[2] Nieman LK (2015). Treatment of Cushing's syndrome: An endocrine society clinical practice guideline. J. Clin. Endocrinol. Metab..

[3] Dickerman RD, Oldfield EH (2002). Basis of persistent and recurrent Cushing disease: An analysis of findings at repeated pituitary surgery. J. Neurosurg..

[4] Katznelson L (2014). Acromegaly: An endocrine society clinical practice guideline. J. Clin. Endocrinol. Metab..

[5] Salenave S (2004). Pituitary magnetic resonance imaging findings do not influence surgical outcome in adrenocorticotropin-secreting microadenomas. J. Clin. Endocrinol. Metab..

[6] Di Maio S, Biswas A, Vézina JL, Hardy J, Mohr G (2012). Pre- and postoperative magnetic resonance imaging appearance of the normal residual pituitary gland following macroadenoma resection: Clinical implications. Surg. Neurol. Int..

[7] Sun H (2014). Factors associated with biochemical remission after microscopic transsphenoidal surgery for acromegaly. J. Neurol. Surg. B Skull Base.

[8] Batista D (2005). Detection of adrenocorticotropin-secreting pituitary adenomas by magnetic resonance imaging in children and adolescents with Cushing disease. J. Clin. Endocrinol. Metab..

[9] Patronas N (2003). Spoiled gradient recalled acquisition in the steady state technique is superior to conventional postcontrast spin echo technique for magnetic resonance imaging detection of adrenocorticotropin-secreting pituitary tumors. J. Clin. Endocrinol. Metab..

[10] Vitale G (2017). Pituitary magnetic resonance imaging in Cushing’s disease. Endocrine.

[11] De Rotte AA (2016). High resolution pituitary gland MRI at 7.0 tesla: A clinical evaluation in Cushing’s disease. Eur. Radiol..

[12] Gao R (2001). Dynamic gadolinium-enhanced MR imaging of pituitary adenomas: Usefulness of sequential sagittal and coronal plane images. Eur. J. Radiol..

[13] Wolfsberger S (2004). Application of three-tesla magnetic resonance imaging for diagnosis and surgery of sellar lesions. J. Neurosurg..

[14] Parrott J, Mullins ME (2005). Postoperative imaging of the pituitary gland. Top. Magn. Reson. Imaging.

[15] Erasmus L, Hurter D, Naudé M, Kritzinger H, Acho S (2004). A short overview of MRI artefacts. SA J. Radiol..

[16] Ogura A, Maeda F, Miyai A, Kikumoto R (2005). Effects of slice thickness and matrix size on MRI for signal detection. Nihon Hoshasen Gijutsu Gakkai zasshi.

[17] Sijbers J, Scheunders P, Bonnet N, Van Dyck D, Raman E (1996). Quantification and improvement of the signal-to-noise ratio in a magnetic resonance image acquisition procedure. Magn. Reson. Imaging.

[18] Plenge E (2012). Super-resolution methods in MRI: Can they improve the trade-off between resolution, signal-to-noise ratio, and acquisition time?. Magn. Reson. Med..

[19] Zhao C (2019). Applications of a deep learning method for anti-aliasing and super-resolution in MRI. Magn Reson Imaging.

[20] Lebel, R. M. Performance characterization of a novel deep learning-based MR image reconstruction pipeline. arXiv preprint arXiv:2008.06559 (2020).

[21] Peters, R. D. & Heide Harris, R. The clinical benefits of AIR™ Recon DL for MR image reconstruction.

[22] Webb SM, Rigla M, Wägner A, Oliver B, Bartumeus F (1999). Recovery of hypopituitarism after neurosurgical treatment of pituitary adenomas. J. Clin. Endocrinol. Metab..

[23] Cordeiro D (2019). Hypopituitarism after Gamma Knife radiosurgery for pituitary adenomas: A multicenter, international study. J. Neurosurg. JNS.

[24] Kucharczyk W, Bishop JE, Plewes DB, Keller MA, George S (1994). Detection of pituitary microadenomas: Comparison of dynamic keyhole fast spin-echo, unenhanced, and conventional contrast-enhanced MR imaging. Am. J. Roentgenol..

[25] Wilson EB (1927). Probable inference, the law of succession, and statistical inference. J. Am. Stat. Assoc..

[26] Dina TS, Feaster SH, Laws ER, Davis DO (1993). MR of the pituitary gland postsurgery: Serial MR studies following transsphenoidal resection. Am J Neuroradiol.

[27] Steiner E (1992). Pituitary adenomas: Findings of postoperative MR imaging. Radiology.

[28] Keen, J. R. & Oyesiku, N. M. in *Complications in Neurosurgery* (ed Nanda, A.) 114–119 (Elsevier, 2019).

[29] Chaudhari AS (2018). Super-resolution musculoskeletal MRI using deep learning. Magn. Reson. Med..

[30] Kim M (2021). Thin-slice pituitary MRI with deep learning-based reconstruction: Diagnostic performance in a postoperative setting. Radiology.

[31] Ilias I (2005). Cushing’s syndrome due to ectopic corticotropin secretion: Twenty years’ experience at the National Institutes of Health. J. Clin. Endocrinol. Metab..

[32] Oldfield EH, Vortmeyer AO (2006). Development of a histological pseudocapsule and its use as a surgical capsule in the excision of pituitary tumors. J. Neurosurg..

[33] Lee EJ (2009). Tumor tissue identification in the pseudocapsule of pituitary adenoma: Should the pseudocapsule be removed for total resection of pituitary adenoma?. Neurosurgery.

[34] Mason RB, Nieman LK, Doppman JL, Oldfield EH (1997). Selective excision of adenomas originating in or extending into the pituitary stalk with preservation of pituitary function. J. Neurosurg..

[35] Tabaee A (2009). Endoscopic pituitary surgery: A systematic review and meta-analysis. J. Neurosurg..

[36] Zhang K, Zuo W, Chen Y, Meng D, Zhang L (2017). Beyond a Gaussian denoiser: Residual learning of deep CNN for image denoising. IEEE Trans. Image Process..

